# Elucidation of Pathophysiology and Novel Treatment for Diabetic Macular Edema Derived from the Concept of Neurovascular Unit

**DOI:** 10.31662/jmaj.2020-0022

**Published:** 2020-07-07

**Authors:** Yoshihiko Usui

**Affiliations:** 1 Department of Ophthalmology, Tokyo Medical University, Tokyo, Japan

**Keywords:** neurovascular units, Retinal neurons, amacrine neurons, diabetic macular edema

## Abstract

The retina transmits light signals to the brain via a complex structure composed of photoreceptor cells, neurons including ganglion cells, glial cells such as astrocytes and Mueller cells, as well as retinal blood vessels that feed the retina. The retina performs such high-level physiological function and maintains homeostasis effectively through interactions among the cells that form the neurovascular units (NVUs). Furthermore, as a component of the blood‒retinal barrier (BRB), the vascular structure of the retina is functionally based on the NVUs, in which the nervous system and the vascular tissues collaborate in a mutually supportive relationship. Retinal neurons such as ganglion cells and amacrine cells are traditionally considered to be involved only in visual function, but multiple functionality of neurons attracted attention lately, and retinal neurons play an important role in the formation and function of retinal blood vessels. In other words, damage to neurons indirectly affects retinal blood vessels. Diabetic macular edema is the leading cause of vision loss in diabetic retinopathy, and this type of edema results in neurological and vascular disorders. In this article, the regulatory mechanism of retinal capillaries in diabetic macular edema is reviewed from the viewpoint of NVU.

## Introduction

**Figure 1.**
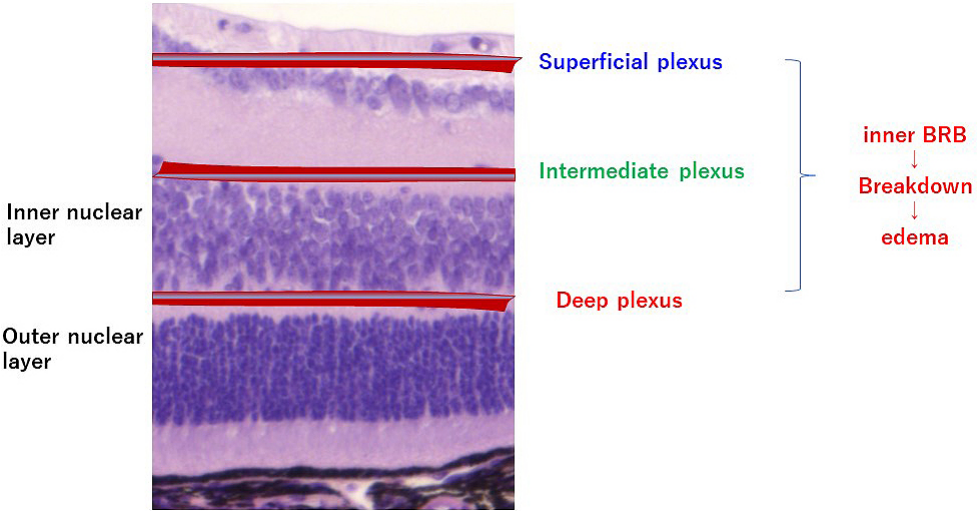
Retinal blood vessels. Retinal capillaries form three major layers. The intermediate capillary plexus is formed between the inner plexiform layer and inner nuclear layer, and the deep capillary plexus is formed in the outer plexiform layer. The retinal capillaries constitute the inner blood retinal barrier (BRB). Breakdown of the inner BRB causes edema.

**Figure 2.**
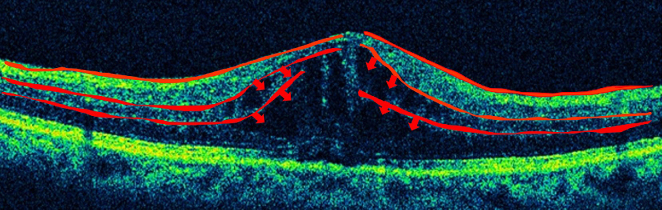
Diabetic macular edema. Edema presumably arises from the capillaries in the intermediate and deep plexuses of the retina, which constitute the inner BRB.

In Japan, more than 12,505 people are estimated to become adventitiously blind every year, and diabetic retinopathy, affecting one-third of the patients with diabetes, is the most common causative disease ^(1), (2)^. Diabetic macular edema is the leading cause of visual impairment in diabetic retinopathy, and current treatments are not adequately effective. Edema results from diverse causes, and it involves various factors such as physical and mechanical factors represented by the vitreomacular traction syndrome, as well as factors associated with inflammation such as vascular endothelial growth factor (VEGF). The macula and retina possess defense mechanisms against inflammation and immune reactions, and the blood‒retinal barrier (BRB) is a representative mechanism. The BRB is composed of an inner barrier and an outer barrier; they are responsible for selectively and actively transporting two-thirds and one-third, respectively, of substances across retinal capillaries to extracellular tissues. The inner BRB maintains homeostasis by preserving normal functions of the capillaries in the retina (Figure 1), and its structure is based on neurovascular units (NVUs) that demonstrate a mutually supporting relationship between the nervous system and the vascular system. In diseases such as diabetic macular edema, retinal vein obstruction, and uveoretinitis, plasma components accumulate in the retina due to BRB breakdown or external factors promoting BRB breakdown, ischemia, hyperglycemia, inflammation, oxidative stress, neuronal death, or inflammatory cytokines secreted due to the above conditions. Since no retinal blood vessel in the macular fovea with a diameter of 0.35 mm exists, the question arises whether macular edema is caused by breakdown of the inner BRB. However, as shown in Figure 2, retinal capillaries traverse even the avascular region of the macular fovea. If leakage occurs from such blood vessels, as shown by the arrow, there is no doubt that edema can occur from retinal blood vessels. Furthermore, since the macula depends on choroidal blood supply, the influence from choroidal circulation is anticipated, and failure of the outer BRB may also play a role in macular edema ^(3)^. This article reviews the relationship between the BRB and the mechanisms that causes edema in the macula and retina, focusing on the NVUs.

## 1. Edema Caused by Increased Vascular Hyperpermeability via Retinal Capillaries and BRB Breakdown

Although the timing of retinal blood vessel formation differs between mice and humans, the formation and distribution of retinal blood vessels are similar. Retinal blood vessels mainly form three layers, the superficial vascular plexus, intermediate vascular plexus, and deep vascular plexus, and these three layers of vascular networks are inter-connected, feeding the intra-retinal tissues. Retinal vascular endothelium is lined by fenestrated and non-fenestrated endothelial cells. Retinal capillaries do not possess adventitia or media but consist of a single layer of vascular endothelial cells. Pericytes are embedded in the perivascular space, and the basement membrane around and between the vascular endothelial cells and pericytes forms the lumen of the blood vessel. The vascular permeability of BRB is regulated by junctions between endothelial cells and binding between endothelial cells and extracellular matrix. For the vascular endothelial cell-cell junction, tight junction is established via tight junction proteins including occludin, VE-cadherin, and claudin present in the intercellular space. In addition, the mechanism ensures that transcellular transport hardly occurs and that the substances inside blood do not pass readily into tissues. As a result, passage of most of the soluble components in blood and immune cells including leukocytes is blocked. In diabetic retinopathy, degeneration and loss of vascular endothelial cells and pericytes, as well as thickening of basement membrane, occur. As a result, the blood vessel wall becomes fragile, and the BRB breaks down, leading to edema. Macular edema is caused by an increase in vascular permeability, due to breakdown of the inner BRB and leakage of intravascular plasma components into the extracellular space. In diabetic macular edema, the inner nuclear layer and outer plexiform layer are more susceptible to edema ^(4)^.

## 2. What is a Neurovascular Unit?

**Figure 3.**
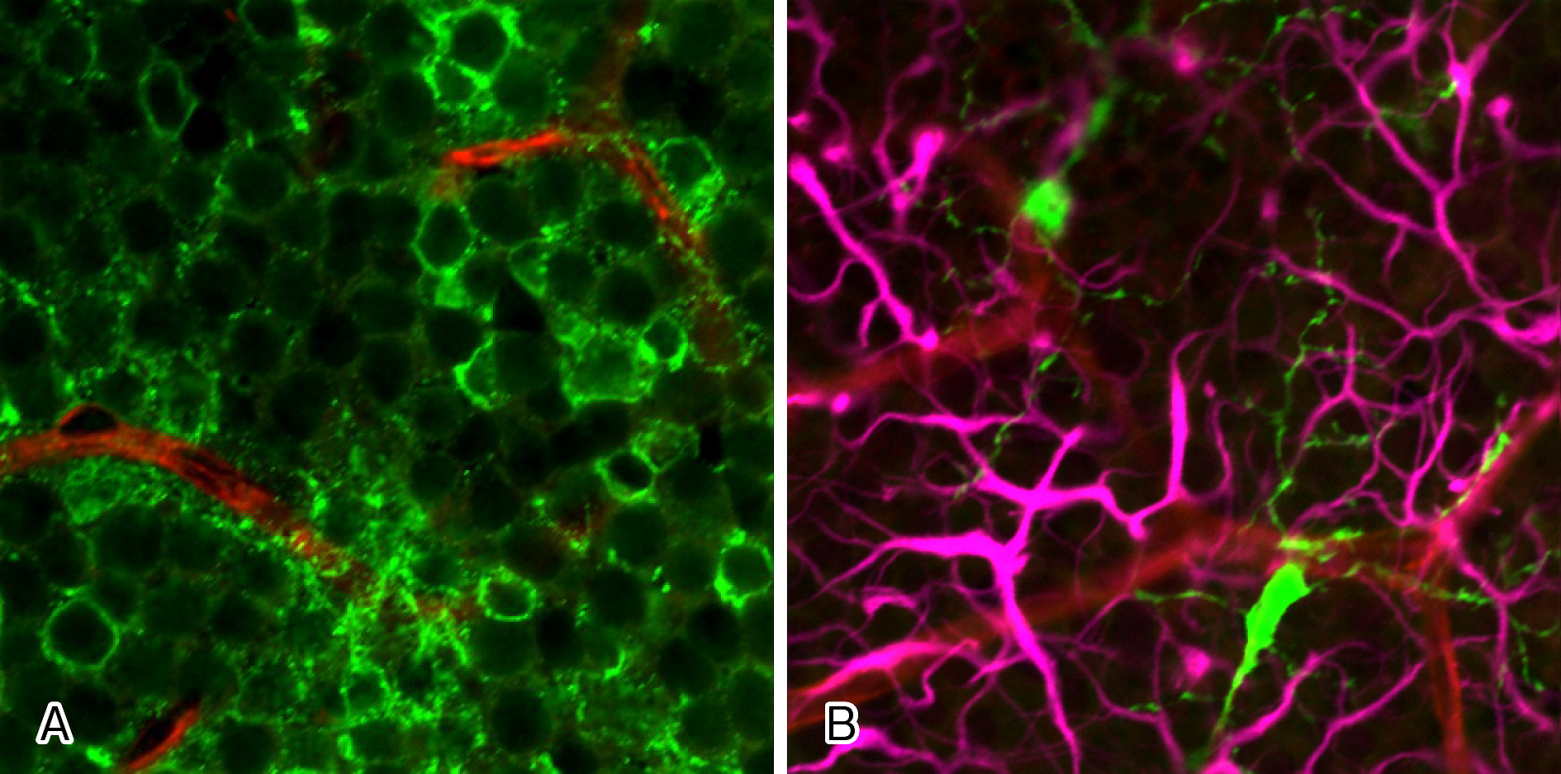
Neurites of retinal amacrine cells, microglia, and capillaries of intermediate plexus of retina. (A) Neurites (green) of numerous amacrine cells (green) are in contact with the capillaries of the intermediate plexus (red). (B) Retinal microglia (green) are in contact with the capillaries of the intermediate plexus (red) and with neurites of amacrine cells (pink).

In the retina, vascular endothelial cells and pericytes of the capillaries, together with neurons, astrocytes, Mueller cells, and glial cells, such as microglia adjoining the capillaries, form a unit, and crosstalk between these cells regulates various functions of the retina. In mouse retina, astrocytes and glial cells such as microglia form the NVUs in the superficial capillary plexus, while neurites of amacrine cells and glial cells such as microglia form the NVUs in the intermediate plexus (Figure 3), and neurites of horizontal cells wrapped by microglia form the NVUs in the deep plexus. Researchers presume that each of the nerves and the adjoining capillaries together control vascular permeability in some way.

## 3. Blood‒retinal Barrier and Neurovascular Unit

**Figure 4.**
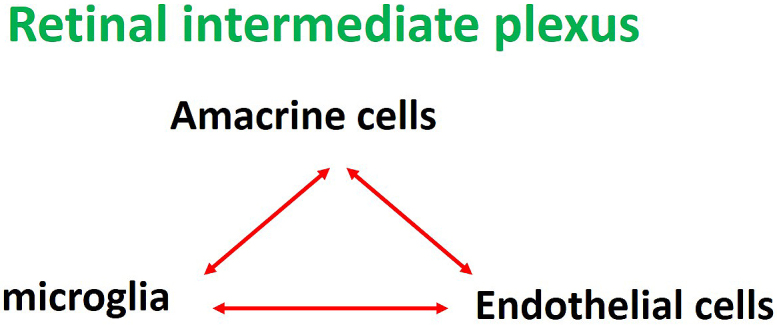
Neurovascular units in the intermediate plexus of retina. Retinal amacrine cells, microglia, and vascular endothelial cells constitute the neurovascular units in the intermediate plexus of the retina.

Researchers have known for more than 20 years that glial cells affect the functions of the BRB ^(5)^, although more recent studies revealed that not only glial cells but neurons also play a role. Neurons such as ganglion cells and amacrine cells were originally thought to be involved only in visual function, but they are now known to be important cells constituting the NVUs. Therefore, neuronal damage may indirectly affect retinal blood vessels and glial cells, and researchers speculate that all the cells constituting the NVUs are involved in BRB breakdown in some ways. The capillaries in the intermediate and deep plexuses of the retina are the main capillaries that constitute the inner BRB. In the intermediate plexus of the retina, amacrine cells and microglia are the major cells that form the NVUs both structurally and functionally, as mentioned above ^(6), (7)^. In other words, the three components, amacrine cells, microglia and capillaries of the intermediate plexus, form the NVUs as a part of the inner BRB (Figure 4).

## 4. Blood‒retinal Barrier Breakdown and Diabetic Macular Edema

**Figure 5.**
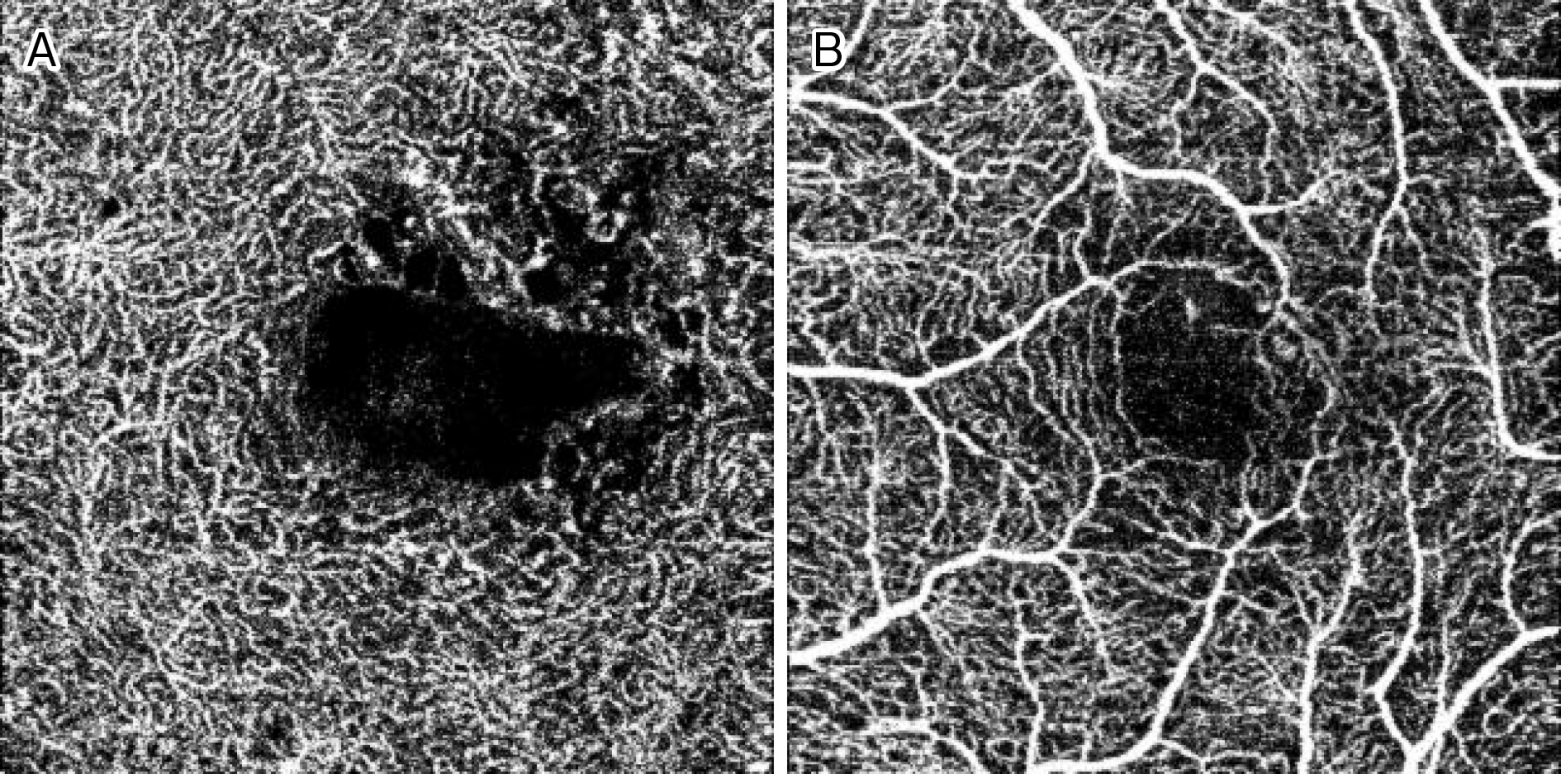
OCT angiography images (Topcon, DRI OCT Triton SSOCT AngioTM, 3×3 mm) of diabetic macular edema. The retinal capillaries in the intermediate or deep plexuses (A), but not superficial plexuses (B), are partially lost.

Among the above-mentioned eye diseases that cause damage to BRB and NVU, diabetic macular edema will be described as an example. In diabetic retinopathy, oscillatory potential (OP) originating from amacrine cells is impaired from an early stage ^(8)^, and abnormal OP has been confirmed also in diabetic model eyes. This OP originated from the amacrine cells present in the inner layers of the retina ^(9)^. Findings of various studies suggested that amacrine cells are impaired and capillaries in the inner layers of the retina are also injured in diabetic retinopathy. These findings include thinning of the inner nuclear layer where amacrine cells are present and the inner plexiform layer where neurites of amacrine cells accumulate in diabetic retinopathy ^(10), (11), (12), (13)^; reduction of blood flow in the retinal capillaries from before the onset of diabetic retinopathy ^(14)^; decreased blood flow in retinal capillaries of the parafoveal region in macular edema ^(15)^; and OCT angiography demonstrating impairment of amacrine cells and damage of retinal capillaries in the deep plexus in diabetic retinopathy (Figure 5) ^(16), (17), (18), (19), (20), (21)^.

**Figure 6.**
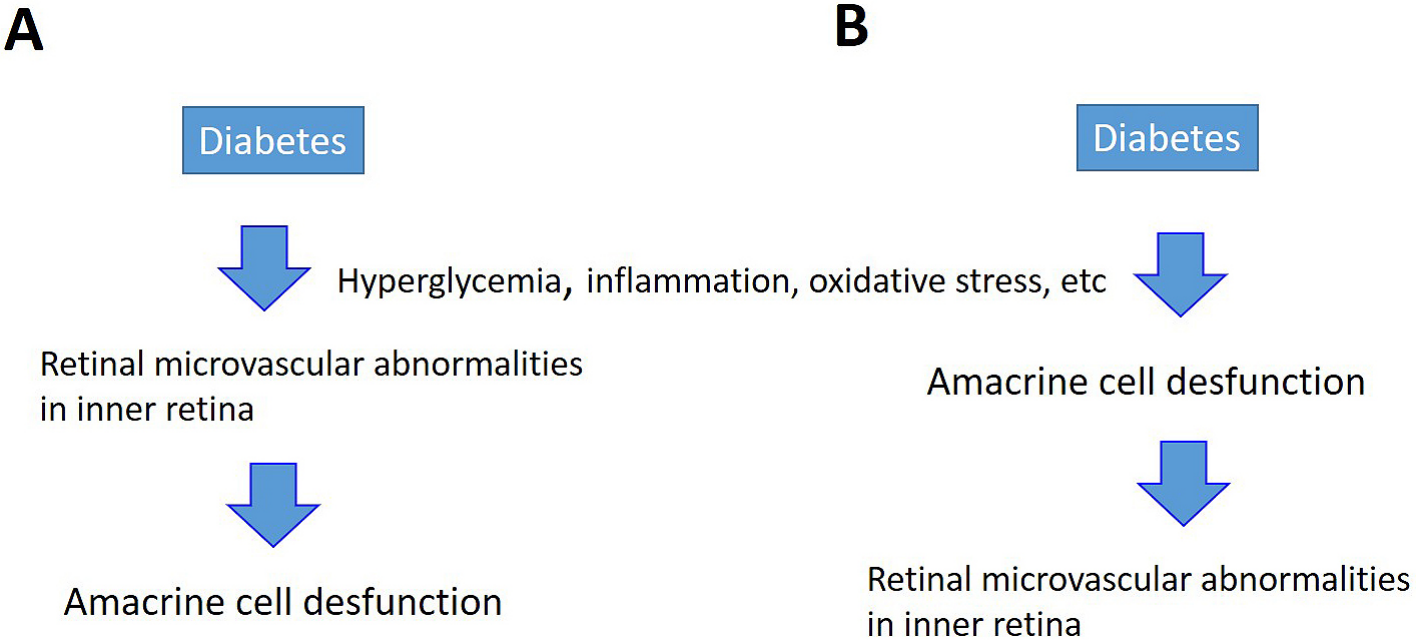
Diabetes-induced damages to blood vessels and amacrine cells in the inner layers of retina. Two pathways are speculated. (A) Hyperglycemia, inflammation, and oxidative stress directly damage capillaries in the retina. (B) Primary damage to neurons that control capillaries in the retina results in secondary damage to retinal vascular endothelial cells.

If the capillaries in the inner layers of the retina are damaged, breakdown of the inner BRB causing edema would occur, as described above. In addition, hard exudates are observed in simple diabetic retinopathy without macular edema. Since hard exudates are proteins or lipids from blood that leaked from retinal blood vessels, they are evidence that vascular permeability increases due to BRB breakdown before macular edema develops. Researchers know that capillaries in the retina are directly damaged by hyperglycemia, inflammation, and oxidative stress. From the viewpoint of the NVU, speculating the existence of an alternate pathway in which neurons that control capillaries in the retina (amacrine cells in the case of diabetic retinopathy) are primarily damaged by the above-mentioned conditions is possible, and then retinal vascular endothelial cells become secondarily damaged (Figure 6). However, since macular edema does not occur in all patients with diabetic retinopathy, the pathological mechanisms require further elucidation through basic research.

## 5. Macular Edema and Cytokines Centered on VEGF

Anti-VEGF agents show certain efficacy for various types of macular edema, including diabetic macular edema and retinal vein occlusion, indicating that VEGF is involved in the pathology of macular edema. VEGF not only promotes division of vascular endothelial cells but also enhances vascular permeability. The mechanism of action has been reported to be activation of the protein kinase C pathway through phosphorylation of occludin that is localized at the tight junction ^(22)^. Most of the cells that constitute the retina (including neurons, glial cells, vascular endothelial cells, and pericytes) produce VEGF, and amacrine cells that control the capillaries in the intermediate plexus of retina also produce VEGF ^(6), (7)^. While edema is reduced by intravitreal injection of anti-VEGF antibody, edema is not totally resolved in many cases, suggesting the involvement of mechanisms other than VEGF. Indeed, high concentrations of other cytokines including IL-8, MCP-1, IP-10, and Mig were detected in the vitreous humor of diabetic retinopathy ^(23), (24)^ and IL-8 and Mig in ischemic retinopathy ^(25)^. These humoral factors may induce inflammation, promote chemotaxis and infiltration of inflammatory cells, and disrupt the BRB ^(26)^. Activated inflammatory cells express cell adhesion factors and secrete matrix metallopeptidases (MMPs) that possess the function of degrading extracellular matrix proteins, thereby destroying the basement membrane and vascular endothelial cells that constitute the BRB. In particular, MMP-9 is speculated to degrade not only the tight junction proteins occludin and claudins but also type IV collagen that constitutes the basement membrane ^(27)^. In addition, apart from MMPs, inflammatory cytokines such as TNF-α, IFN-γ, and IL-1 can also disrupt the BRB ^(28)^ When the BRB is disrupted, infiltration of inflammatory cells from the damaged site is facilitated. In addition, microglia adjoining the endothelial cells also respond to external stimuli and promptly elicit inflammatory responses ^(29)^. However, in intraocular inflammatory diseases such as uveitis, although macular edema may occur as the main inflammation, not all types of uveoretinitis cause macular edema. The prevalence of macular edema is the highest in ocular sarcoidosis and Behcet disease but is low in Vogt-Koyanagi-Harada disease (data not shown). These findings suggest that macular edema due to BRB breakdown is not simply due to inflammatory cytokines. 

Furthermore, VEGF demonstrates a neuroprotective effect. From the viewpoint of neurovascular protection, studies showed that continuous administration of anti-VEGF antibodies for age-related macular degeneration causes atrophy of the retina ^(30)^, and that in diabetic macular edema, anti-VEGF agents are less effective in patients with lower density of capillaries in the intermediate or deep plexus of the retina. These phenomena can be explained rationally by adopting the concept of the NVU. 

In this review, we highlighted the current understanding of how the neurons and vascular systems interact to ensure proper diabetic macular edema. With the advent of anti-VEGF agents, a paradigm shift occurred in the treatment of diabetic macular edema and macular edema due to retinal vein occlusion, but the treatment effect for macular edema remains unsatisfactory. One of the reasons is that focus is currently placed on treatments that target the retinal capillaries, despite the fact that the cause is microcirculatory failure due to breakdown of the inner BRB: that is, disruption of NVUs consisting of both the retinal capillaries and the nervous system. In diabetic macular edema, dysfunction of neurons such as amacrine cells is speculated to cause macular edema. This aspect requires further study as a potential target for new drug development, and aiming at restoration of the damaged functional network of glial cells and neurons adjoining retinal blood vessels (neurovascular remodeling) is necessary. For this reason, research and development from a complex perspective are required, and the final endpoint is likely to be a long way away. Since research with an integrated concept of NVU in the retina started not so long ago, many new findings did not pass the speculative stage, and instead, they await further validation. Nevertheless, the notion that macular edema arises from dysfunction of NVUs is a rational hypothesis.

An advanced high-throughput approach such as proteomic, transcriptomic, and metabolomic technologies may apply, to extend our understanding of molecular changes in NVU in the retina and to provide potential biomarkers for diabetic macular edema, highlighting the importance of future research in this field of ophthalmology.

## Article Information

### 

This article is based on the study, which received the Medical Research Encouragement Prize of The Japan Medical Association in 2019.

### Conflicts of Interest

Yoshihiko Usui received research grants from Bayer Yakuhin, Ltd.

### Sources of Funding

This work was supported by Grand-in-Aid for Scientific Research (C) grant numbers 16K11330, 19K09981, and 19K09959 from the Ministry of Education, Culture, Sports, Science and Technology of Japan, Research grants from Public Interest Foundation for the Elderly Eye Diseases Research Foundation.

### Author Contributions

Conceptualization and design of the study was conducted by Y.U. The experiments were performed and analyzed by Y.U. The original draft of the manuscript was prepared by Y.U. Funding acquisition was conducted by Y.U.

### Approval by Institutional Review Board (IRB)

None
